# How Socio-Environmental Factors Are Associated with Japanese Encephalitis in Shaanxi, China—A Bayesian Spatial Analysis

**DOI:** 10.3390/ijerph15040608

**Published:** 2018-03-27

**Authors:** Shaobai Zhang, Wenbiao Hu, Xin Qi, Guihua Zhuang

**Affiliations:** 1 School of Public Health, Xi’an Jiaotong University, Xi’an 710061, China; maolyzhang@126.com; 2 Department of Immunization Program, Shaanxi Center for Disease Control and Prevention, Xi’an 710054, China; 3 School of Public Health and Social Work, Queensland University of Technology, Kelvin Grove, QLD 4059, Australia; w2.hu@qut.edu.au; 4 Global Health Institute, Xi’an Jiaotong University, Xi’an 710061, China; chestertsee@outlook.com

**Keywords:** Japanese encephalitis, meteorological variables, contingent risk factors, Shaanxi of China, geographical information system

## Abstract

Evidence indicated that socio-environmental factors were associated with occurrence of Japanese encephalitis (JE). This study explored the association of climate and socioeconomic factors with JE (2006–2014) in Shaanxi, China. JE data at the county level in Shaanxi were supplied by Shaanxi Center for Disease Control and Prevention. Population and socioeconomic data were obtained from the China Population Census in 2010 and statistical yearbooks. Meteorological data were acquired from the China Meteorological Administration. A Bayesian conditional autoregressive model was used to examine the association of meteorological and socioeconomic factors with JE. A total of 1197 JE cases were included in this study. Urbanization rate was inversely associated with JE incidence during the whole study period. Meteorological variables were significantly associated with JE incidence between 2012 and 2014. The excessive precipitation at lag of 1–2 months in the north of Shaanxi in June 2013 had an impact on the increase of local JE incidence. The spatial residual variations indicated that the whole study area had more stable risk (0.80–1.19 across all the counties) between 2012 and 2014 than earlier years. Public health interventions need to be implemented to reduce JE incidence, especially in rural areas and after extreme weather.

## 1. Introduction

Japanese encephalitis (JE) is a mosquito-borne disease caused by infection with Japanese encephalitis virus (JEV) that causes an infection of the brain [1]. The major clinical symptoms of human infected JE cases are fever, headaches, nausea and vomiting, loss of consciousness, movement disorders, seizures, and acute flaccid paralysis [2,3]. *Culex* mosquitoes, pigs and paddling birds compose the enzootic circle of JE transmission [1,4]. Usually, rural areas have many *Culex tritaeniorhynchus*, the primary vector species of the JE host, and mosquitoes can reproduce and proliferate in wet areas, e.g., flood areas, rice paddies and grasses [1]. Mosquitoes can transmit JEV and infect humans, the dead-end host of JE infection [5]. Currently, 24 countries in the Asian and Western Pacific regions (with a combined population of over three billion) have a significant JE burden, with around 68,000 estimated JE cases occurring annually [2,6]. With socioeconomic development, improved living conditions, clinical diagnosis and treatment improvement, vaccination and substantial vector control programs, JE incidence has been significantly reduced in some countries, e.g., China, Japan and South Korea [7,8,9,10].

In general, areas in Asian and Western Pacific regions with lower latitude and higher temperature and rainfall had higher JE incidence [2,6]. Recent studies showed a significant variety of JE incidences across different areas in China, with high risk areas in the southwest of the country [11,12,13,14]. Other studies examined the impact of climate factors (e.g., rainfall and temperature) on JE in China and other countries over time and space, as spatiotemporal variation of meteorological factors can influence the mosquito population distribution [9,15,16]. Wang et al. examined the association of meteorological and other environmental variables with JE presence in the whole of China [11], however, this study did not include human population data across different areas in China and cannot present how socio-environmental variables affects the JE incidence across areas with different population distributions [11]. Besides, China has a variety of climate zones and significant socio-environmental and demographical status disparity across the country. National studies reported very brief results in examining the socio-environmental impact on JE. Thus, understanding the spatiotemporal pattern and relevant socio-environmental factors of JE in a relative smaller study area (e.g., using county level data in a province of China) may provide more specific and precise evidence for local JE control and prevention. With the progress of global climate change, the impacts of extreme weather and natural disasters (e.g., flood resulted from excessive precipitation) on mosquito-borne diseases have become more prominent, including areas usually with low incidence (e.g., Taiwan) [17,18]. JE incidence has a significant seasonality, with a peak from late spring to early autumn, or in the wet season [19,20,21]. Thus, understanding the dynamic pattern of JE incidence and examining relevant socio-climate factors may help preventing resurgence of JE epidemic in areas with low incidence. This study used the JE surveillance data (2006–2014) in Shaanxi Province, China, to quantify how meteorological and socio-demographical factors associated with human JE incidence at the county level, to identify risk factors of JE, and to provide evidence for JE control and prevention strategies, especially in the early warning and forecasting system development. 

## 2. Materials and Methods

### 2.1. Study Site

Shaanxi Province (longitudes: 105°29′ to 110°15′ E, latitudes: 31°42′ to 39°35′ N) lies in the middle of China, covering an area of 205,600 square kilometers with a population of 37,327,378 (according to the Sixth National Census of China in 2010). The province consists of 107 county level districts (abbreviated as countiesr) with distinct climate zones: subtropical and humid climate in the south, warm temperate climate in the middle, and temperate and dry climate in the north. 

### 2.2. Data Collection

JE data from between 2006 and 2014, including gender, age, county and township code of the home address, date of symptom emergence and hospital admission, and the annual data of JE vaccination doses (2006–2014), were acquired from Shaanxi Provincial Center for Disease Control and Prevention (CDC). Meteorological data (monthly), including rainfall, temperature (maximum, minimum and mean), humidity, sunshine and air pressure, were supplied by the China Meteorological Administration. The original meteorological data were at the station level with the longitude and latitude of all 86 observational stations (36 stations in Shaanxi and 50 stations in the neighbouring provinces) applied in this study. Then we used Kriging interpolation to transfer the station level data into the county level data. Socio-demographical data at the county level, including human population by age and gender, urbanization rate (percentage of urban population among the total population), domestic pig population and mean elevation, were from China Population Census in 2010 and statistical yearbooks. Since the domestic pig population is associated with human population size across areas, especially in rural areas, we also calculated the pig to human population ratio for further analysis. As Shaanxi has a relative lower population growth rate than the national level since 2000, we used the population data in 2010 to represent the population during the whole study period. 

### 2.3. Data Analyses

In this study, we defined JE incidence at the county level as the dependent variable, which was calculated after adjusting for gender and age. Climate and socio-demographical variables were defined as independent variables. As JE has a relative lower incidence in Shaanxi compared to the national level, we firstly aggregated the JE data into three 3-year intervals in the study period: 2006–2008, 2009–2011 and 2012–2014, for displaying spatial pattern of JE incidence in maps. This helps presen a more stable spatial pattern of JE incidence. Besides, the annual spatial pattern of JE incidence was also mapped. Nine-year JE incidence data, monthly meteorological variables and yearly socio-demographical data at the county level were shown. The annual doses of JE vaccination at the provincial level was also plotted. Spearman correlation analysis was conducted to demonstrate the correlations between JE incidence and socio-environmental variables and test the multicollinearity among the independent variables. 

Usually, analysis of JE incidence at the small areas (e.g., at the county level) with a small number of cases accrued annually in most areas may cause unstable and biased results in estimating local incidence and factors associated with JE incidence. To solve this problem, spatial smoothing can be applied to enhance quality of estimates using “borrowed strength” from neighbouring small areas if we assume these areas having common characteristics. A Bayesian Conditional Autoregressive (CAR) model can provide a local smoothing approach to reduce the bias of small area data analysis with few cases and sparse population [22]. Besides, the spatial autocorrelation, which is usually localized and varies across different areas, needs to be included in the models to enhance estimates of the associations of socio-environmental factors and JE incidence. Hence, mapping the variation of spatial correlation residuals may explain other factors which may associated with geographical difference of JE incidence. The Bayesian CAR model has been widely used in examining the impact of social and climate variables on public health, including infectious disease [23], non-communicable disease [24,25] and injury [26]. We used the Bayesian CAR model with Poisson distribution to analyze the association of socio-environmental variables with JE incidence at the county level. The formulation of Bayesian CAR model is as follows [27]:

log(*μ_i_*) = log(*n_i_*) + (*β*_0_ + *β*_1_*X*_1*i*_ + … + *β_m_X_mi_*) + *U_i_* + *S_i_*

where *U_i_*(the unstructured random effects) and *S_i_* (the structured random effects) represent a Poisson distribution with mean *μ_i_* in above formula; *i*, *n*, *μ_i_*, *X* and *β* indicates location, population, the mean of the dependent variable (JE incidence), the fixed effect and socio-environmental variables, respectively. *β*_0_ + *β*_1_*X*_1*i*_ + … + *β_m_X_mi_* shows the regression equation. These random effects are spatially correlated. We firstly analyzed three sets of aggregated datasets (3-year for each) of JE cases, population and independent variables separately using Besag, York and Mollie (BYM) models indicating structured and unstructured residuals. This explored the differences of associations of socio-environmental variables with JE incidence across three periods. We checked the deviance information criterion (DIC) to detect the goodness of fit in the Bayesian CAR model, the lower DIC the better. In the first stage, we conducted preliminary analyses without spatial elements in the Bayesian CAR model. Then we continuously added covariance (*U_i_*) and structured covariance (*S_i_*) in the model, showing the residuals of the final model at the county level in the map. As JE incidence has seasonality, both of the aggregated yearly data (in Model I) and data from selected months with high incidence (in Model 2) were applied in the analyses. To avoid the multicollinary problem, we checked the high correlations among predictor variables. (*r_s_* ≥ |0.80|). We applied the Markov Chain Mount Carlo (MCMC) simulation in estimating parameter and diagnosis, using single chain algorithm. All types of models were burnt-in with 30,000 iterates and run for 90,000 iterates. Autocorrelations of selected parameters were used to check the convergence. The Bayesian CAR model was run by the WinBUGS package. This project used aggregated data and the Ethics Committee of Xi’an Jiaotong University Health Science Center provided the required ethical approval. 

## 3. Results

**Figure 1:**
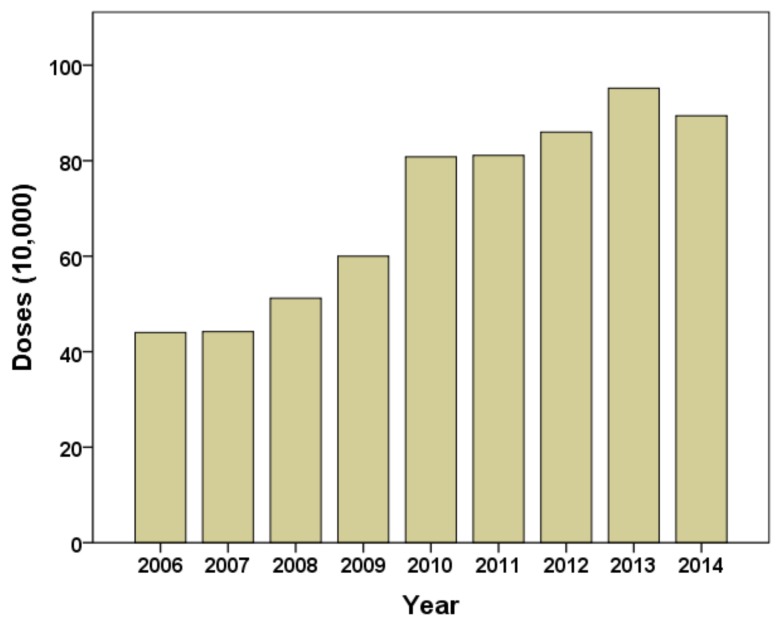
Annual distribution of JE vaccination doses at the provincial level (2006–2014).

This study included a total of 1197 JE cases (2006–2014) of which 99.7% (1193 cases) of the total cases occurred in summer and early autumn (between June and October), as Table 1 shows. JE cases peaked in August each year. The middle region had over half of the JE cases; while the north had the lowest number of JE cases each year except for 2013. Around 35.7% of total JE cases occurred in 006. In general, JE cases had a decrease trend in the whole study period. The annual distribution of JE vaccination doses has increased since the vaccination program started in 2006 (Figure 1). The urban counties (with 60% and more urban population), sub-urban counties (with 30% to 59% urban population) and rural counties (with less than 30% of urban population) had annual average JE incidence of 0.14, 0.41 and 0.44 per 100,000, respectively. 

**Figure 2:**
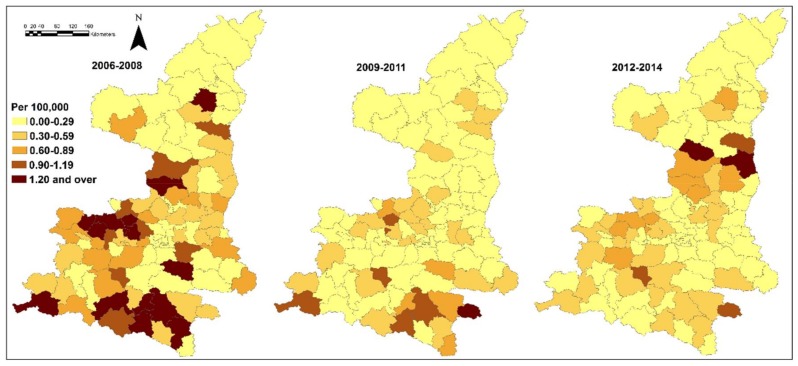
Japanese encephalitis (annual average per 100,000) in Shaanxi, China (2006–2014).

Table 2 indicates the spatial distribution of JE incidence and socio-environmental variables selected at the county level in this study. Meteorological variables and JE incidence covered the months between June and October 2006–2014. Most variables had an approximately normal distribution. Figure 2 shows the JE incidence (annual average) at the county level in three study periods. In general, 2006–2008 had a higher incidence than 2009–2014. The south, west of the middle and part of the north had the highest incidence between 2006 and 2008. Areas with high incidence were also discovered in the south between 2009 and 2011, and in the north between 2012 and 2014. The spatial pattern of JE incidence in each year was mapped in Supplementary File Figure S1. The years 2006, 2007, 2009 and 2013 had a significant spatial variation of JE incidence. 

Table 3 demonstrated the correlations between JE incidence and socio-environmental variables over the whole study period and across counties. Rainfall (RF), humidity (HM), air pressure (AP) and pig to human ratio (PHR) were positively correlated with JE incidence, while sunshine (SS), urbanization rate (UB) and population density (PPD) had inverse correlations with JE incidence. Other variables had weak correlation with JE incidence. 

Based on the above correlation results and variables associated with JE from the literature, we selected RF, Tmin, UB, PPD and PHR, and examined their associations with JE incidence in the Bayesian CAR model. The posterior estimates of the results were shown as mean value and standardized division (SD). Table 4 summarized the association of socio-environmental variables with JE incidence over different study periods (Model 1). UB was inversely associated with JE incidence in the whole study period. RF was positively associated with JE incidence, and Tmin had inverse association with JE incidence between 2012 and 2014. The study period from 2006 to 2008 had much higher DIC value than following 6 years. We also tested the associations of HM, Tmax, Tmean, SS, AP and EL with JE incidence in the three study periods using similar model and found weaker results. 

**Figure 3:**
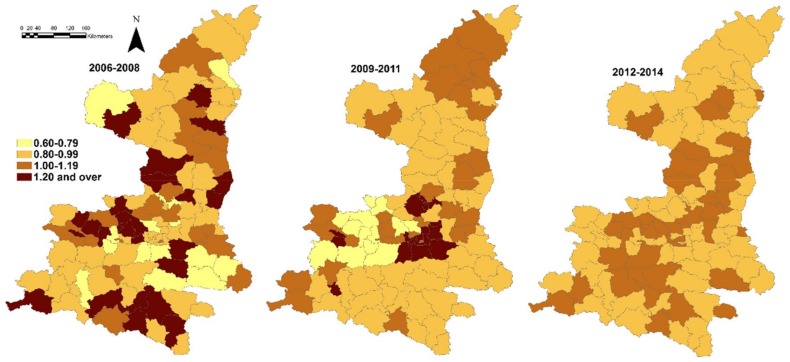
Spatial random effect for JE incidence (2006–2014), structured spatial residuals with fixed effect in Model I.

The spatial residual variations (the structured random effects) of Model I were mapped in Figure 3; and socio-environmental variables were considered in the model. The spatial residual variations were presented as relative risk (RR) in the same study period. 

Between 2006 and 2008, there were still distinct spatial residual variation across counties; and high-risk areas (RR ≥ 1.20) were distributed evenly in the whole study area. A cluster of high risk areas in the middle of Shaanxi was also apparent in the period from 2009 to 2011. During the last three-year period (2012–2014), all the counties had stable RR ranging from 0.80 to 1.19. In general, the spatial random effects had a more heterogeneous distribution than spatial distribution of incidence of the same period, especially in the last period. We also found higher DIC value in models with no added random effect index, besides the model we applied to obtain the results above. 

The pattern of JE incidence between 2012 and 2014 was different from that in earlier years of this study. Meteorological variables had more significant associations with JE incidence in this period than previous years. No JE case was discovered in the north in 2012 and 2014 (Table 1), and the north area had much higher proportion of JE cases in the whole province in 2013 (22.36%) than the average of the whole study period (6.93%), especially in August (11 of 88 total cases, or 12.50%) and September (18 of 44 total cases, or 40.91%). Thus, we assumed that some extreme weather event(s) (e.g., excessive rainfall) may have resulted in high incidence in the north in 2013. Then the association of meteorological variables and socioeconomic factors (UB, PPD and PHR) with JE incidence in August and September 2013 with 1 to 3-month lag was examined (Model II). Table 5 indicated that RF was inversely associated with JE incidence in August and September with three months lag each (rainfall in May and June) but had positive association with JE incidence in September with two months lag (rainfall in July). 

There was an inverse association between Tmin and JE incidence in September with 1 and 3-month lag (Tmin in June and August). UB and PHR were inversely associated with JE incidence in August. The spatial residual of Model II in 2013 (all types of lag effect) had stable relative risk ranging from 0.96 to 1.07. The DIC value in Model II was much lower than Model I. We also applied Model II in other years with relative high incidence (e.g., 2006 and 2009), and found no significant association between meteorological factors and JE incidence. 

## 4. Discussion

This study explored the pattern of JE incidence and examined socio-environmental impact on JE in Shaanxi, China (2006–2014). The spatial pattern of JE incidence and significant socio-environmental drivers of JE incidence changed across three periods. In the third period, meteorological variables were more significantly associated with JE incidence than previous years. There was significant lag effect of impact of meteorological factors on JE incidence in August and September 2013. The results from this study may help researchers to have a better understanding of JE in local areas, and provide precise evidence for establishing and conducting targeted and more effective strategies on JE control and prevention to public health policy makers and practitioners.

Meteorological variables can change the living and proliferation environment of mosquitoes, and lead to variation of mosquito density over time and space. This may influence the development and transmission of virus from mosquito to human population and other hosts [28,29]. Precipitation can leave a humid environment, which can foster the survival, growth and reproduction of mosquitoes [30,31,32]. Thus, the increased humidity can add the transmission of JEV from mosquito to susceptible hosts, including humans. The subtropical south of Shaanxi, composed of mountains, rivers and valleys, has a humid environment and around 25 °C mean temperature in summer, which provide a suitable environment for mosquito reproduction and JEV transmission, especially in areas near rivers. In most of the study period, the south region had higher precipitation than the middle and north of Shaanxi. This may explain why JE incidence was higher in the south in most of the study period. However, during the whole month of July 2013, heavy rainfall occurred in the north of Shaanxi with around 500 mm precipitation, which was higher than the annual average local precipitation, and was also higher than precipitation in the middle and south that month. In our study, the two months lag for rainfall had a significant and positive association with JE in September 2013, which is consistent with previous studies [13,33]. Other literature has also indicated that temperatures over 21 °C are suitable for mosquito population growth and increase of JE incidence over time in tropical and subtropical regions [19,33,34]. A suitable temperature helps mosquito larva and JEV development, and can foster the spread of mosquitoes and JE disease [11,35]. In general, areas with lower latitude in Shaanxi had higher temperature in each month in the whole study period. In our study, Tmin were inversely associated with JE incidence in Model I (2012–2014) and Model II (September 2013, 3 months lag), indicating that the geographical distribution of rainfall (especially during excessive precipitation period) may have a higher impact on the spatial variation of JE incidence than other meteorological variables (e.g., temperature). The north of Shaanxi, which was traditionally regarded as a relatively dry area with low incidence of mosquito-borne diseases, may have relative weaker adaptation capacity to disease outbreak resulted from extreme weather than other areas. All above may explain that the spatial pattern of JE incidence in August and September 2013 was different from that in the remaining study period.

The progress of urbanization, which may devastate the suitable environment for mosquito population survive and growth, have been accompanied with decrease of JE incidence in Eastern Asia [7,8,9,10]. In Shaanxi, the middle region (including Xi’an, the provincial capital) has a higher urbanization level than the north and south. Thus, fewer high risk areas were discovered in the middle of Shaanxi than the south and the north in general; and areas with higher urbanization level had lower JE incidence than the remaining areas in the middle of the province. JEV can be transmitted from pigs to humans by mosquito biting [5]. The pig to human ratio in the south of Shaanxi was over 150% higher than the middle and north respectively in each year of the whole study period. Our study found that PHR was only inversely associated with JE incidence in Model II (2013), which is different from other studies [13,36]. The results in this study indicated that urbanization rate and extreme weather (e.g., rainfall) may have more significant impact on the spread of JEV and spatial variation of JE incidence than PHR and other variables in this study. The distinct spatial pattern of JE incidence in August and September, 2013 (higher in the north than middle and south) and PHR resulted in the inverse association, from statistical perspective. Other spatial studies using point data indicated that land cover types and ground vegetation can also influence the distribution of mosquito [5,37,38,39]. In Shaanxi, most of farms and crop lands are in the rural areas, especially in the middle and south; and the south has more bodies of water. This may be associated with geographical distribution of JE cases in most years of the study period. However, our study used administrative geographical units in data analysis, which is difficult in demonstrating the details of types of land use and vegetation at the county level, including snow melt and the distance to bodies of water of each JE case. Evidence indicated that immunization programs have reduced JE incidence in China over time [9,10]. The reduction of JE incidence in Shaanxi, especially in the south (a traditionally high-risk area), may be attributed to the implementation of immunization programs in these areas. However, the impact of immunization program on JE at the county level cannot be examined in this study due to the unavailability of dynamic vaccination information at the county level. The dynamitic mosquito population data at the county are not available in this study. Thus, the impact of mosquito population and relevant key factors (e.g., type of land use, vegetation and snow melt) on JE cannot be examined. 

The covariates and random effects applying in spatial autocorrelation adjustment, and borrowed strength from adjacent areas in the Bayesian CAR model, can reduce the randomly created risk likelihood in spatial analysis [40]. In this study, JE annual average incidence rate between 2012 and 2014 were lower than earlier years in this study. And the spatial residual variation in this period was more homogenous than previous years. The Bayesian CAR model indicated that other factors, e.g., vaccination program, healthcare facilities, land types, may result in the spatial variation of residuals and have more significant impact on the geographical difference of JE incidence in earlier years. Bayesian CAR model has also been used in other studies in infectious and non-communicable diseases [26,41,42].

There are a few strengths in this study. Firstly, this is the first study exploring the association of meteorological and socio-demographical variables with JE incidence in the study site with a relative low JE disease burden in China, using a Bayesian spatial model. Secondly, this study used two types of spatial models: Model I provided a relative stable pattern of JE incidence using aggregated yearly data and examined the impact of socio-environmental factors on JE incidence. Model II discovered that extreme weather (e.g., excessive precipitation) can change the spatial pattern of JE incidence with lag effect and had higher impact on mosquito-borne disease than other variables, using monthly data. The comparison of two models provided more in-depth understanding of spatial pattern of JE and relevant factors. Finally, the results of this study can provide more precise evidence for public health implication on local JE control and prevention programs than previous national studies, focusing on high risk areas and significant socio-environmental factors. 

However, some weaknesses should also be addressed. Firstly, some factors which can influence the spread of JEV, e.g., personal health behavior, vaccination at the county level, mosquito population over time and at the county level, types of land use and vegetation at the county level, are not available or not applicable using administrative geographical units in our spatial analysis. Secondly, some cases without county information were not included in this study, and this may result in a bias underestimating JE incidence in some areas.

## 5. Conclusions

In conclusion, the association between socio-environmental variables and JE incidence has become more significant in the recent years compared to earlier years in Shaanxi (China). Rainfall and urbanization rate were the most significant factors associated with JE. The impact of progressing climate change and more frequent extreme weather occurrence on mosquito-borne disease need to be notified and future public health implication on JE control and prevention, especially in rural areas. Areas traditionally having low incidence and prevalence of mosquito-borne disease need to enhance capacity of adapting to climate change and its impact on resurgence of infectious disease. 

## Figures and Tables

**Table 1:** Distribution of JE cases (2006–2014) by month and region in Shaanxi, China.

	Years									Total
	2006	2007	2008	2009	2010	2011	2012	2013	2014	
Months										
January	0	0	0	0	0	0	0	0	0	0
February	1	0	0	0	0	1	0	0	0	2
March	1	0	0	0	0	1	0	0	0	2
April–May	0	0	0	0	0	0	0	0	0	0
June	2	0	0	1	2	0	0	0	0	5
July	134	10	1	37	10	4	0	13	0	209
August	322	92	31	116	65	17	48	88	49	828
September	11	17	10	8	24	5	15	44	6	140
October	1	1	0	0	1	1	0	7	0	11
November–December	0	0	0	0	0	0	0	0	0	0
Regions										
North	31	3	2	9	4	0	0	34	0	83
Middle	253	67	25	85	67	18	46	92	30	683
South	188	50	15	68	31	11	17	26	25	431
Total	472	120	42	162	102	29	63	152	55	1197

**Table 2:** JE incidences and social and -climate variables in Shaanxi, China (2006–2014).

	Mean	SD.	Min	Quantile			
				25	50	75	Max
IN	0.37	0.32	0.00	0.12	0.29	0.55	1.55
RF	95.33	21.00	58.74	83.08	88.03	105.06	169.68
HM	72.07	5.57	59.02	69.93	71.45	75.46	81.53
Tmax	25.91	0.93	23.49	25.30	25.78	26.50	28.08
Tmin	20.17	1.59	12.01	14.78	16.05	17.02	18.60
Tmean	15.82	1.12	17.96	19.18	20.16	21.04	22.38
SS	174.95	24.57	129.24	158.30	171.19	185.29	233.27
AP	916.76	19.58	870.95	901.31	916.33	931.42	957.59
UB	41.19	19.95	17.17	28.44	33.36	47.27	100.00
PPD	10.13	36.26	0.18	0.78	1.65	4.67	270.70
PHR	0.39	0.38	0.00	0.13	0.27	0.51	2.28
EL	738.88	300.47	212.00	469.00	693.00	959.00	1543.00

Note: IN (incidence, per 100,000), RF (rainfall, mm), HM (humidity, %), Tmax (maximum temperature, °C), Tmin (minimum temperature, °C), Tmean (mean temperature, °C), SS (sunshine, hours), AP (air pressure, kpa), UR (urbanization rate, %), PPD (population density, per km^2^), PHR (pig to human ratio, per 10,000 persons), EL (mean elevation, m). Monthly mean data of IN and meteorological variables, yearly mean data of UB, PPD and PHR.

**Table 3:** Spearman correlations of JE incidences and socio-climate variables in Shaanxi, China (2006–2014).

	IN	RF	HM	Tmax	Tmin	Tmean	SS	AP	UB	PPD	PHR
RF	0.415 **	1.000									
HM	0.326 **	0.907 **	1.000								
Tmax	0.109	0.450 **	0.516 **	1.000							
Tmin	0.171	0.653 **	0.780 **	0.883 **	1.000						
Tmean	0.150	0.561 **	0.680 **	0.929 **	0.982 **	1.000					
SS	−0.220 *	−0.763 **	−0.906 **	−0.545 **	−0.782 **	−0.708 **	1.000				
AP	0.162	0.579 **	0.673 **	0.945 **	0.963 **	0.986 **	−0.685 **	1.000			
UB	−0.414 **	−0.334 **	−0.240 *	−0.072	−0.124	−0.103	0.112	−0.133	1.000		
PPD	−0.212 *	−0.186	0.021	0.117	0.259 **	0.292 **	−0.204 *	0.242 *	0.230 *	1.000	
PHR	0.284 **	0.544 **	0.474 **	0.414 **	0.439 **	0.409 **	−0.380 **	0.422 **	−0.437 **	−0.351 **	1.000
EL	0.035	−0.113	−0.306 **	−0.526 **	−0.621 **	−0.643 **	0.422 **	−0.603 **	−0.122	−0.679 **	−0.001

Note: ** *p* ≤ 0.01, * *p* ≤ 0.05, IN (incidence), RF (rainfall), HM (humidity), Tmax (maximum temperature), Tmin (minimum temperature), Tmean (mean temperature), SS (sunshine), AP (air pressure), UB (urbanization rate), PPD (population density), PHR (pig to human ratio), EL (mean elevation). Monthly mean data of IN and meteorological variables, yearly mean data of UB, PPD and PHR.

**Table 4:** Meteorological, socio-economic factors and JE incidences (Model I: three-year aggregated data).

Year	Variable	Mean	SD	MC Error	2.50%	Median	97.50%
2006–2008 DIC: 482.357	RF	−0.00505	0.026	0.001346	−0.05675	−0.00562	0.04801
	Tmin	0.1757	0.8161	0.04699	−1.392	0.2904	1.381
	**UB**	**−0.01447**	**0.006339**	**8.7 × 10^−5^**	**−0.02694**	**−0.01446**	**−0.00206**
	PPD	−0.00505	0.005126	5.15 × 10^−5^	−0.01562	−0.00489	0.004593
	PHR	−0.3778	0.3247	0.008936	−1.017	−0.3775	0.259
2009–2011 DIC: 381.612	RF	−0.002776	0.01136	4.55 × 10^−4^	−0.02498	−0.002908	0.01965
	HM	0.0434	0.09243	0.005146	−0.1153	0.03623	0.2319
	Tmin	1.59	1.488	0.0858	−1.496	1.411	4.751
	**UB**	**−0.01466**	**0.007114**	**1.28** × **10^−4^**	**−0.029**	**−0.01457**	**−9.74** × **10^−4^**
	PPD	−0.005596	0.00557	5.00 × 10^−5^	−0.0174	−0.005294	0.004608
	PHR	−0.7085	0.4878	0.01128	−1.677	−0.7072	0.2421
2012–2014 DIC: 401.925	**RF**	**0.003558**	**0.001654**	**6.26 × 10^−5^**	**5.81** × **10^−4^**	**0.003453**	**0.007144**
	**Tmin**	**−3.901**	**1.248**	**0.0719**	**−6.03**	**−3.89**	**−1.489**
	**UB**	**−0.02025**	**0.005439**	**8.26 × 10^−5^**	**−0.03087**	**−0.02026**	**−0.009464**
	PPD	−0.001663	0.004063	5.48 × 10^−5^	−0.01047	−0.001387	0.00552
	PHR	−0.4917	0.2732	0.006128	−1.051	−0.4817	0.01762

Note: RF (rainfall) Tmin (minimum temperature), UB (urbanization rate), PPD (population density), PHR (pig to human ratio). The bold indicates variables significantly associated with JE incidence.

**Table 5:** Meteorological, socio-economic factors and JE incidences (Model II: 2013).

Month of JE	Lag and DIC	Variable	Mean	SD	MC Error	2.50%	Median	97.50%
August	1-month DIC: 252.858	RF	0.004155	0.002431	8.09 × 10^−5^	−7.73 × 10^−4^	0.004221	0.008774
		Tmin	−1.083	1.174	0.06731	−4.112	−0.8657	0.6437
		**UB**	**−0.02564**	**0.009119**	**1.21 × 10^−4^**	**−0.0438**	**−0.02556**	**−0.007921**
		PPD	−0.01713	0.01472	1.12 × 10^−4^	−0.05335	−0.01435	0.003748
		**PHR**	**−1.165**	**0.5232**	**0.01226**	**−2.261**	**−1.141**	**−0.209**
	2-month DIC: 251.518	RF	0.00312	0.007369	2.84 × 10^−4^	−0.01214	0.003419	0.01692
		Tmin	−2.779	1.397	0.08016	−5.123	−3.009	0.2639
		**UB**	**−0.02573**	**0.00924**	**1.20 × 10^−4^**	**−0.04408**	**−0.02565**	**−0.00777**
		PPD	−0.01325	0.01315	1.37 × 10^−4^	−0.04547	−0.01084	0.005502
		**PHR**	**−0.9929**	**0.5402**	**0.01096**	**−2.15**	**−0.9573**	**−0.03795**
	3-month DIC: 243.944	**RF**	**−0.05158**	**0.01391**	**5.44 × 10^−4^**	**−0.07931**	**−0.05139**	**−0.02518**
		Tmin	0.2289	0.952	0.05355	−1.685	0.3494	1.904
		**UB**	**−0.02759**	**0.008652**	**1.45 × 10^−4^**	**−0.04498**	**−0.02749**	**−0.01094**
		PPD	−0.01212	0.01335	1.65 × 10^−4^	−0.045010	−0.009631	0.006669
		**PHR**	**−1.664**	**0.5583**	**0.01578**	**−2.83**	**−1.634**	**−0.6456**
September	1-month DIC: 155.262	RF	−0.04816	0.02992	0.00103	−0.1111	−0.04633	0.005663
		**Tmin**	**−2.952**	**1.387**	**0.07904**	**−5.973**	**−2.87**	**−0.05671**
		UB	−0.01319	0.01744	2.87 × 10^−4^	−0.04756	−0.0133	0.02167
		PPD	−0.1252	0.1036	0.00244	−0.3847	−0.1015	0.002195
		PHR	−0.3601	0.753	0.0169	−1.856	−0.3579	1.128
	2-month DIC: 140.323	**RF**	**0.01925**	**0.004357**	**2.08 × 10^−4^**	**0.01163**	**0.0189**	**0.02872**
		Tmin	−1.68	2.081	0.1196	−5.456	−1.376	1.871
		UB	−0.01898	0.01402	1.76 × 10^−4^	−0.04658	−0.01895	0.008462
		PPD	−0.1021	0.08615	1.70 × 10^−^^3^	−0.3129	−0.08403	0.006762
		PHR	−0.07627	0.5582	0.01121	−1.267	−0.04381	0.9268
	3-month DIC: 147.809	**RF**	**−0.05426**	**0.02199**	**9.76 × 10^−4^**	**−0.09975**	**−0.05336**	**−0.01424**
		**Tmin**	**−5.791**	**1.562**	**0.08906**	**−9.645**	**−5.492**	**−3.489**
		UB	−0.02307	0.01343	1.76 × 10^−4^	−0.05014	−0.02288	0.002658
		PPD	−0.06881	0.0592	8.42 × 10^−4^	−0.2163	−0.05536	0.00457
		PHR	−0.5815	0.5584	0.009745	−1.767	−0.5504	0.4278

Note: RF (rainfall) Tmin (minimum temperature), UB (urbanization rate), PPD (population density), PHR (pig to human ratio). The bold indicates variables significantly associated with JE incidence.

## Supplementary Materials

The following are available online at http://www.mdpi.com/1660-4601/15/4/608/s1, Figure S1: The spatial pattern of JE incidence in each year.

## References

[1] SAGE Working Group Background Paper on JE Vaccines. http://www.who.int/immunization/sage/meetings/2014/october/1_JE_Vaccine_Background_Paper.pdf.

[2] Ghosh D., Basu A. (2009). Japanese encephalitis-a pathological and clinical perspective. PLoS Negl. Trop. Dis..

[3] Misra U.K., Kalita J. (2010). Overview: Japanese encephalitis. Prog. Neurobiol..

[4] Van den Hurk A.F., Ritchie S.A., Mackenzie J.S. (2009). Ecology and geographical expansion of Japanese encephalitis virus. Annu. Rev. Entomol..

[5] Solomon T. (2006). Control of Japanese encephalitis—Within our grasp?. N. Engl. J. Med..

[6] Campbell G.L., Hills S.L., Fischer M., Jacobson J.A., Hoke C.H., Hombach J.M., Marfin A.A., Solomon T., Tsai T.F., Tsui V.D. (2011). Estimated global incidence of Japanese encephalitis: A systematic review. Bull. World Health Organ..

[7] Gao X., Nasci R., Liang G. (2010). The neglected arboviral infections in mainland China. PLoS Negl. Trop. Dis..

[8] Erlanger T.E., Weiss S., Keiser J., Utzinger J., Wiedenmayer K. (2009). Past, present, and future of Japanese encephalitis. Emerg. Infect. Dis..

[9] Pan X.F., Griffiths U.K., Pennington M., Yu H., Jit M. (2015). Systematic review of economic evaluations of vaccination programs in mainland China: Are they sufficient to inform decision making?. Vaccine.

[10] Li X., Gao X., Ren Z., Cao Y., Wang J., Liang G. (2014). A spatial and temporal analysis of Japanese encephalitis in mainland China; 1963–1975: A period without Japanese encephalitis vaccination. PLoS ONE.

[11] Wang L., Hu W., Soares Magalhaes R.J., Bi P., Ding F., Sun H., Li S., Yin W., Wei L., Liu Q. (2014). The role of environmental factors in the spatial distribution of Japanese encephalitis in mainland China. Environ. Int..

[12] Wang L.Y., Zhang W.Y., Ding F., Hu W.B., Soares Magalhaes R.J., Sun H.L., Li Y.X., Zou W., Wang Y., Liu Q.Y. (2013). Spatiotemporal patterns of Japanese encephalitis in China; 2002–2010. PLoS Negl. Trop. Dis..

[13] Zhao X., Cao M., Feng H.H., Fan H., Chen F., Feng Z., Li X., Zhou X.H. (2014). Japanese encephalitis risk and contextual risk factors in southwest China: A Bayesian hierarchical spatial and spatiotemporal analysis. Int. J. Environ. Res. Public Health.

[14] Li X., Cui S., Gao X., Wang H., Song M., Li M., Fu S., Lv Z., He Y., Lei W. (2016). The Spatio-temporal Distribution of Japanese Encephalitis Cases in Different Age Groups in Mainland China, 2004–2014. PLoS Negl. Trop. Dis..

[15] Impoinvil D.E., Ooi M.H., Diggle P.J., Caminade C., Cardosa M.J., Morse A.P., Baylis M., Solomon T. (2013). The effect of vaccination coverage and climate on Japanese encephalitis in Sarawak; Malaysia. PLoS. Negl. Trop. Dis..

[16] Impoinvil D.E., Solomon T., Schluter W.W., Rayamajhi A., Bichha R.P., Shakya G., Caminade C., Baylis M. (2011). The spatial heterogeneity between Japanese encephalitis incidence distribution and environmental variables in Nepal. PLoS ONE.

[17] Zhang F., Liu Z., Zhang C., Jiang B. (2016). Short-term effects of floods on Japanese encephalitis in Nanchong; China; 2007–2012: A time-stratified case-crossover study. Sci. Total. Environ..

[18] Chen M.J., Lin C.Y., Wu Y.T., Wu P.C., Lung S.C., Su H.J. (2012). Effects of extreme precipitation to the distribution of infectious diseases in Taiwan, 1994–2008. PLoS ONE.

[19] Lin C.L., Chang H.L., Lin C.Y., Chen K.T. (2017). Seasonal Patterns of Japanese Encephalitis and Associated Meteorological Factors in Taiwan. Int. J. Environ. Res. Public Health.

[20] Lee H.S., Nguyen-Viet H., Lee M., Duc P.P., Grace D. (2017). Seasonality of Viral Encephalitis and Associated Environmental Risk Factors in Son La and Thai Binh Provinces in Vietnam from 2004 to 2013. Am. J. Trop. Med. Hyg..

[21] Samuel P.P., Arunachalam N., Rajendran R., Leo S.V., Ayanar K., Balasubramaniam R., Tyagi B.K. (2010). Temporal variation in the susceptibility of *Culex tritaeniorhynchus* (Diptera: Culicidae) to Japanese encephalitis virus in an endemic area of Tamil Nadu; South India. Vector Borne Zoonotic Dis..

[22] Escaramís G., Carrasco J.L., Ascaso C. (2008). Detection of significant disease risks using a spatial conditional autoregressive model. Biometrics.

[23] Gou F., Liu X., Ren X., Liu D., Liu H., Wei K., Yang X., Cheng Y., Zheng Y., Jiang X. (2017). Socio-ecological factors and hand; foot and mouth disease in dry climate regions: A Bayesian spatial approach in Gansu; China. Int. J. Biometeorol..

[24] Wang Y., Kockelman K.M. (2013). A Poisson-lognormal conditional-autoregressive model for multivariate spatial analysis of pedestrian crash counts across neighborhoods. Accid. Anal. Prev..

[25] Tassone E.C., Waller L.A., Casper M.L. (2009). Small-area racial disparity in stroke mortality: An application of bayesian spatial hierarchical modelling. Epidemiology.

[26] Qi X., Hu W., Mengersen K., Tong S. (2014). Socio-environmental drivers and suicide in Australia: Bayesian spatial analysis. BMC Public Health.

[27] Pfeiffer D., Robinson T., Stevenson M., Stevens K.B., Rogers D., Clements A.C. (2008). Spatial Analysis in Epidemiology.

[28] Balenghien T., Carron A., Sinègre G., Bicout D.J. (2010). Mosquito density forecast from flooding: Population dynamics model for *Aedes caspius* (Pallas). Bull. Entomol. Res..

[29] Hay S.I., Myers M.F., Burke D.S., Vaughn D.W., Endy T., Ananda N., Shanks G.D., Snow R.W., Rogers D.J. (2000). Etiology of interepidemic periods of mosquito-borne disease. Proc. Natl. Acad. Sci. USA.

[30] Masuoka P., Klein T.A., Kim H.C., Claborn D.M., Achee N., Andre R., Chamberlin J., Small J., Anyamba A., Lee D.K. (2010). Modeling the distribution of *Culex tritaeniorhynchus* to predict Japanese encephalitis distribution in the Republic of Korea. Geospat. Health.

[31] Le Flohic G., Porphyre V., Barbazan P., Gonzalez J.P. (2013). Review of climate; landscape; and viral genetics as drivers of the Japanese encephalitis virus ecology. PLoS Negl. Trop. Dis..

[32] Miller R.H., Masuoka P., Klein T.A., Kim H.C., Somer T., Grieco J. (2012). Ecological niche modeling to estimate the distribution of Japanese encephalitis virus in Asia. PLoS Negl. Trop. Dis..

[33] Bi P., Zhang Y., Parton K.A. (2007). Weather variables and Japanese encephalitis in the metropolitan area of Jinan city, China. J. Infect..

[34] Murty U.S., Rao M.S., Arunachalam N. (2010). The effects of climatic factors on the distribution and abundance of Japanese encephalitis vectors in Kurnool district of Andhra Pradesh; India. J. Vector Borne Dis..

[35] Zheng Y., Li M., Wang H., Liang G. (2012). Japanese encephalitis and Japanese encephalitis virus in mainland China. Rev. Med. Virol..

[36] De A., Maity K., Jana S., Maiti M. (2016). Application of various control strategies to Japanese encephalitic: A mathematical study with human; pig and mosquito. Math Biosci..

[37] Sarkar A., Patil S., Hugar L.B., vanLoon G. (2011). Sustainability of current agriculture practices; community perception; and implications for ecosystem health: An Indian study. Ecohealth.

[38] Raju K.H., Sabesan S., Rajavel A.R., Subramanian S., Natarajan R., Thenmozhi V., Tyagi B.K., Jambulingam P. (2016). A Preliminary Study to Forecast Japanese Encephalitis Vector Abundance in Paddy Growing Area; with the Aid of Radar Satellite Images. Vector Borne Zoonotic Dis..

[39] Sunish I.P., Reuben R. (2001). Factors influencing the abundance of Japanese encephalitis vectors in rice fields in India—I. Abiotic. Med. Vet. Entomol..

[40] Lee D. (2011). A comparison of conditional autoregressive models used in Bayesian disease mapping. Spat. Spatio-Temporal Epidemiol..

[41] Alegana V.A., Atkinson P.M., Wright J.A., Kamwi R., Uusiku P., Katokele S., Snow R.W., Noor A.M. (2013). Estimation of malaria incidence in northern Namibia in 2009 using Bayesian conditional-autoregressive spatial-temporal models. Spat. Spatio-Temporal. Epidemiol..

[42] Hu W., Clements A., Williams G., Tong S., Mengersen K. (2010). Bayesian spatiotemporal analysis of socio-ecologic drivers of Ross River virus transmission in Queensland; Australia. Am. J. Trop. Med. Hyg..

