# Supplementary figures and images for: How Socio-Environmental Factors Are Associated with Japanese Encephalitis in Shaanxi, China—A Bayesian Spatial Analysis
Source: Int J Environ Res Public Health. 2018 Mar 27;15(4):608. doi: 10.3390/ijerph15040608 (PMC5923650; doi:10.3390/ijerph15040608)

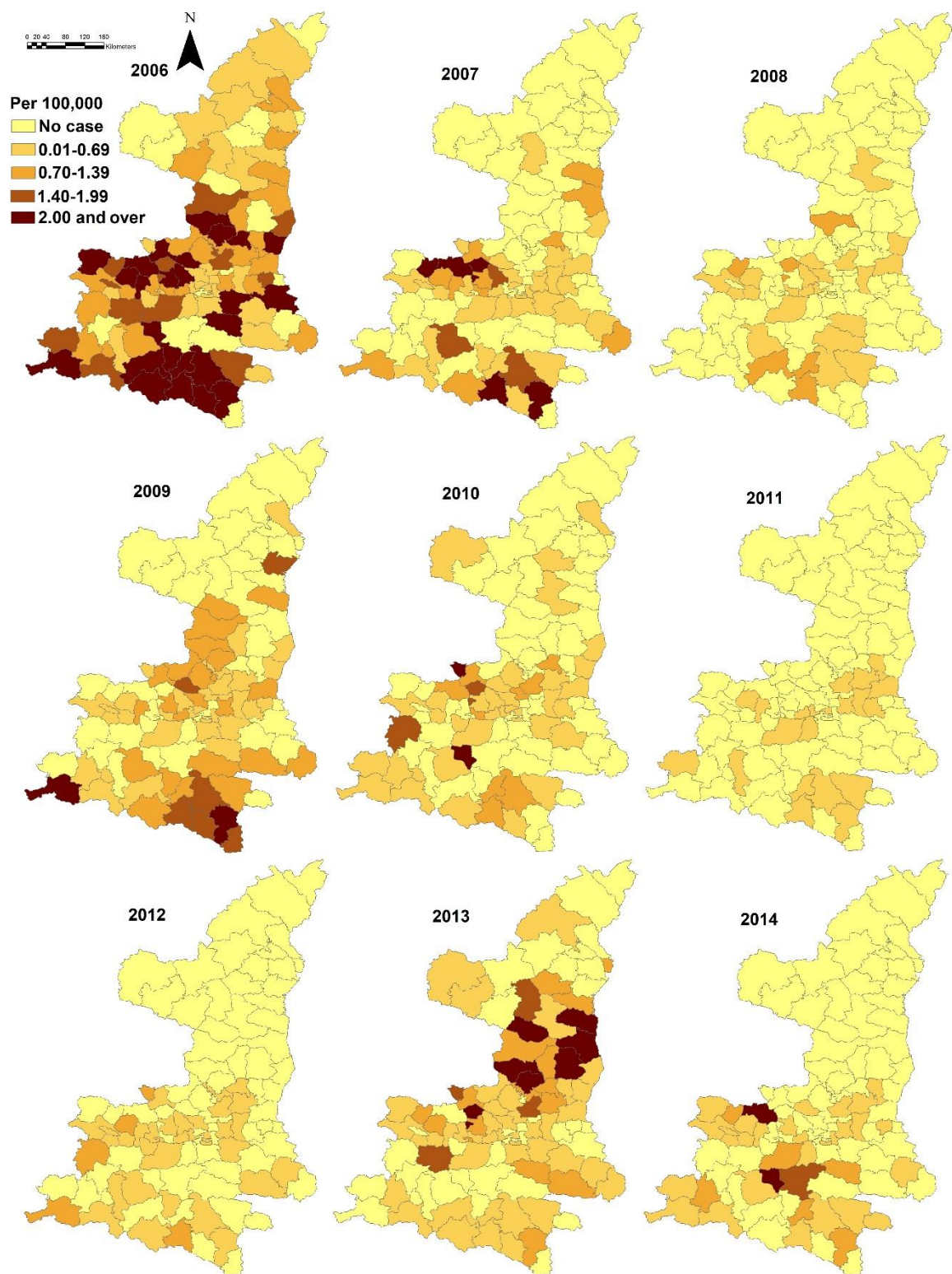

**Figure S1.** The spatial pattern of JE incidence in each year.

Supplement: Supplementary file 1 [file ijerph-15-00608-s001.pdf]
